# Knowledge of Complications of Diabetes Mellitus Among Patients Visiting the Diabetes Clinics: A Cross-Sectional Study in the Qassim Region

**DOI:** 10.7759/cureus.49896

**Published:** 2023-12-04

**Authors:** Ahmad A Alrasheedi, Muath H Alqesair, Hailah F Aljurbua, Ghaida A Alfanoud, Mohammed H Almakrami, Jamal E J. Mohammad

**Affiliations:** 1 Department of Family and Community Medicine, College of Medicine, Qassim University, Buraidah, SAU; 2 Department of Family Medicine, University of Szeged Albert Szent-Györgyi Medical School, Szeged, HUN; 3 Department of Family and Community Medicine, Qassim University, Buraidah, SAU

**Keywords:** family, qassim, knowledge, complications, diabetes, awareness

## Abstract

Background and objectives

Diabetes mellitus (DM) is a chronic complex metabolic disease characterized mainly by hyperglycemia. It appears to be a global epidemic and an increasingly major non-communicable disease, posing a threat to both affluent and nonaffluent societies. Diabetes dramatically increases the risk of developing stroke, chronic kidney disease, and coronary artery disease. These complications include chronic kidney disease, retinopathy, coronary artery disease, stroke, and diabetic foot ulcers. Compared to people without diabetes, adults with diabetes have a two to four times greater risk of dying from heart disease and stroke. This study aimed to assess the knowledge and attitude regarding complications among patients with diabetes visiting the Diabetes Clinics at King Fahad Specialist Hospital (KFSH) in the Qassim Region, Saudi Arabia.

Methodology

A cross-sectional study was conducted in which patients with DM who visited the Diabetic Clinics at KFSH from February 2023 to July 2023 were recruited using a nonprobability sampling technique. A validated questionnaire from previous research was used to collect data on the sociodemographic features of the participants and their knowledge regarding diabetic complications. Data were described using mean with standard deviation for continuous variables and proportion for categorical variables.

Results

A total of 368 patients were recruited. The majority of the respondents (144, 39.1%) had been living with diabetes for over 10 years, followed by 23.1% (85) of those with less than one year. Exactly 239 (64.9%) reported having a family history of diabetes. About 75% (276) of participants knew about diabetic complications, and 247 (67.1%) reported that their doctor had addressed them about diabetes complications. Among the sources of diabetes complication information, diabetologists were reported by 131 (35.6%) of the respondents, followed by 81 (22.0%) through social media, 65 (17.7%) from relatives, and 39 (10.6%) from family medicine doctors. On the other hand, the findings reveal that the different age groups have different levels of knowledge of hyperglycemia and hypoglycemia as diabetic complications (*P *= 0.031). However, there were no significant differences in the level of knowledge regarding complications between male and female patients (*P* > 0.05).

Conclusions

The study found that while the studied population had a relatively good knowledge of diabetic complications, some still lacked knowledge. The study also supports the need for individuals with a family history of diabetes to be aware of their risk and take steps for prevention, as a large proportion of participants reported having a family history of diabetes. Healthcare providers and diabetologists were the most frequent sources of information on diabetic complications. However, some also turned to social media. Focused education and awareness actions are crucial to ensure that people with diabetes have access to reliable information from various sources.

## Introduction

Diabetes mellitus (DM) is a chronic, complex metabolic disease characterized mainly by hyperglycemia [1]. It appears to be a global epidemic and an increasingly major non-communicable disease, posing a threat to both affluent and nonaffluent societies. Diabetes dramatically increases the risk of developing stroke, chronic kidney disease, and coronary artery disease [2].

According to the etiology, there are two forms of DM: type 1 and type 2. Type 1 DM development is significantly influenced by immune system dysregulation; however, its origin is uncertain. According to the literature, the likelihood of acquiring type 1 DM from a mother with the disease is 1% to 4%, whereas the likelihood of acquiring it from a father with it is 3% to 8% [3]. Type 2 DM is a multifactorial disease with a complex etiology involving genetic, metabolic, and environmental factors. Key pathogenic factors include dysregulation of adipokines, inflammation, and alterations in gut flora [4]. Moreover, obesity is a significant factor in the development of type 2 DM because it causes insulin resistance in peripheral tissues, an increase in pro-inflammatory markers, and other metabolic problems, which are all brought on by obesity [5].

If not managed correctly, diabetes complications can affect the majority of the body's organs, particularly the heart, blood vessels, kidneys, eyes, nerves, and teeth. These complications include chronic kidney disease, retinopathy, coronary artery disease, stroke, and diabetic foot ulcers. Compared to people without diabetes, adults with diabetes have a two to four times higher risk of mortality from heart disease and stroke. Moreover, 67% of diabetics in the United States have high blood pressure [6].

Adequate knowledge of diabetes is a key component of diabetic care. Many studies have discussed the depth of knowledge and awareness among patients with diabetes regarding the complications of this serious disease. A survey of 570 participants conducted in the Western Region of Saudi Arabia reported that approximately 76% of the participants were aware of the complications of diabetes as communicated by their treating physician. Compared to their awareness of hypoglycemia symptoms, which was less spectacular, their understanding of hyperglycemia symptoms was fairly good. Various factors, such as residing in Taif, possessing a college degree, and having a family history of diabetes, were found to positively influence knowledge of diabetes complications [7]. According to research of 630 participants, the majority of individuals with diabetes (60%) did not have knowledge of diabetes complications, while 26.9% had inadequate understanding of diabetes complications [8].

In another study on diabetes complications with 96 patients (79% of the patients being illiterate), 37.5% had high knowledge, 25% had moderate knowledge, and 37.5% had low knowledge. Males and patients with a bachelor's degree had somewhat more knowledge [9]. At Jimma University, Ethiopia, a higher percentage of patients with diabetes lacked sufficient knowledge about diabetes complications. The degree of knowledge of diabetes complications was substantially linked with age, male gender, high-income earners, higher education, and urban residency among individuals with both type 1 and type 2 diabetes [6]. Based on a study of 100 participants, about 95% said they knew about diabetic complications from health institutions and about 51% had good knowledge of diabetes complications [10]. While in Bangladesh's capital, Dhaka, the level of knowledge, attitude, and practices were rated 9.2 (out of 14), 7.9 (out of 13), and 16.9 (out of 27), respectively. Age and gender were significant predictors of knowledge and attitude, according to the study [11].

Hence, we hypothesized that patients visiting the diabetic clinics at King Fahad Specialty Hospital (KFSH) in the Qassim Region, Saudi Arabia, have a good awareness of the complications of DM. Hence, the objective of this study was to evaluate the extent of knowledge and awareness concerning complications among patients with diabetes attending the Diabetes Clinics at King Fahad Specialist Hospital in the Qassim Region, Saudi Arabia. The knowledge level regarding diabetic complications between male and female patients was also evaluated.

## Materials and methods

A cross-sectional study was undertaken, encompassing patients aged 18 years or older with DM who attended the Diabetic Clinics at KFSH in Buraydah, Qassim Region, during the period from February 2023 to July 2023. Exclusions were made for diabetic patients with severe complications (such as blindness, end-stage renal disease, diabetic foot, etc.) and pregnant women.

The sample size was estimated to be 351. We assumed that the nonresponse rate would be 5%, so the total number of about 368 participants was estimated to be the requirement for conducting this study. Among all suitable patients, by employing convenient sampling, 368 patients were recruited.

Primary data were collected directly from the participants by a self-administrated questionnaire; a validated questionnaire was taken from previous research [7]. Illiterate individuals can ask someone they trust to help them read and fill out the questionnaire. The study variables encompassed demographic data (gender, age, nationality, social status, educational level, and occupation), information on diabetes mellitus (duration and family history), and whether the healthcare provider had ever discussed complications of diabetes mellitus with the participant. If yes, details were collected regarding the source of this information. Also, the variables included the participants' knowledge about DM complications (general and specific). Data were stored in files, such as Excel files, with access restricted to research team members only.

Statistical analysis was carried out using SPSS, Version 26 (IBM Corp., Armonk, NY). Data were described using mean with standard deviation (SD) for continuous variables and proportion for categorical variables. A chi-square test was performed for categorical variables, while t-tests were performed for continuous variables to assess the difference between the variables according to gender. A *P*-value < 0.05 was considered statistically significant.

Ethical approval was obtained from the Qassim University Research Committee (the institutional review board number was CL-2023010401). The participants had the right to refuse or accept the study to participate in this study based on their benefits. We obtained informed consent from the patients.

## Results

Table 1 presents the demographic characteristics of the participants, categorized by gender. The average age of participants was 39.17 years, with females significantly older than males (39.74 vs. 37.75). The majority of the participants (329, 89.4%) were Saudi. Regarding marital status, 210 (57.1%) were married, followed by 133 (36.1%) individuals who were single. A total of 221 (60.1%) of the respondents had a university degree. The participants had a diverse range of occupations. The largest group consisted of employed individuals, comprising 122 respondents (33.2%). According to gender, there were significant differences in age, marital status, and occupation.

**Table 1:** Demographic characteristics of the participants categorized by gender. ^*^Data presented as *n* (%) or mean ± standard deviation (SD). The *P*-value was considered significant at *P *< 0.05.

Social and clinical characteristics	Total (*n* = 368), *n* (%)	Female (*n *= 266), *n* (%)	Male (*n *= 102), *n* (%)	*P*-value*
Age (18-85 years)	39.17 ± 15.38	39.74 ± 15.40	37.75 ± 15.32	0.015
Nationality				0.129
Non-Saudi	39 (10.6%)	34 (87.2%)	5 (12.8%)	
Saudi	329 (89.4%)	232 (70.5%)	97 (29.5%)	
Marital status				0.004
Divorced	12 (3.3%)	7 (58.3%)	5 (41.7%)	
Married	210 (57.1%)	158 (75.3%)	52 (24.7%)	
Single	133 (36.1%)	88 (66.2%)	45 (43.8%)	
Windowed	13 (3.5%)	13 (100%)	0 (0.0%)	
Education level				0.408
Illiteracy	13 (3.5%)	12 (92.3%)	1 (7.3%)	
Primary	23 (6.3%)	14 (60.9%)	9 (29.1%)	
Secondary	97 (26.4%)	65 (67.0%)	32 (23%)	
Intermediate	14 (3.8%)	10 (71.4%)	4 (28.6%)	
University and above	221 (60.1%)	164 (74.5%)	56 (25.5%)	
Occupation				0.023
Employed	122 (33.2%)	76 (62.3%)	46 (37.7%)	
Freelancer	18 (4.9%)	5 (27.7%)	13 (72.3%)	
Retired	58 (15.8%)	41 (70.7%)	17 (29.3%)	
Student	79 (21.5%)	59 (74.7%)	20 (25.3%)	
Unemployed	91 (24.7%)	85 (93.4%)	6 (6.6%)	

Table 2 presents information on the prevalence, history, and source of DM complication information in the study population. The highest number (144, 39.1%) had been living with diabetes for over 10 years, followed by less than one year (85, 23.1% ). Exactly 239 ( 64.9%) reported having a family history of diabetes. The majority of the participants (247, 67.1%) reported that their doctor had addressed them about diabetes complications. Among the sources of diabetes complication information, diabetologists were reported by 131 (35.6%) of the respondents, followed by 81 (22%) through social media, 65 (17.7%) from relatives, and 39 (10.6%) from family medicine doctors.

**Table 2:** Clinical characteristics and sources of diabetes mellitus complication information among the participants.

Clinical characteristics	Frequency and proportion, *n* (%)
Diabetes duration	
Less than one year	85 (23.1%)
More than 10 years	144 (39.1%)
1-10 years	81 (22.0%)
5-10 years	58 (15.8%)
Family history of diabetes	
Yes	239 (64.9%)
No	129 (35.1%)
Doctors have discussed diabetes complications with you.	
Yes	247 (67.1%)
No	121 (32.9%)
Sources of diabetes complication information	
Books and papers	15 (4.1%)
Diabetologists	131 (35.6%)
Family medicine doctors	39 (10.6%)
Other doctors	14 (3.8%)
Others	20 (5.4%)
Relatives	65 (17.7%)
Social media	81 (22.0%)
Volunteer campaigns	3 (0.8%)

Table 3 shows that exactly 84% (309) of the participants understood that DM could lead to loss of vision. Similarly, exactly 80.2% (295) of the participants were aware that DM could lead to renal disease. Regarding cardiovascular complications, a total of 261 (70.9%) participants knew that DM could lead to heart disease, and 188 (51.1%) understood it can lead to stroke. Further, 254 (69%) participants knew that DM could lead to sexual impairment, and 227 (75.3%) asserted it can lead to peripheral neuropathy. Interestingly, a higher percentage of female participants had a good understanding of most complications compared to male participants. There was a statistically significant difference between loss of vision (*P* = 0.035) and dental problems (*P *= 0.008) based on gender.

**Table 3:** Level of knowledge of specific complications of DM among the participants based on gender. ^*^Number (%) of the participants who have good knowledge. ^#^The chi-square test *P*-value was considered significant if *P *< 0.05. DM, diabetes mellitus

Specific complications of DM	Total (*n *= 368), *n* (%)*	Female, *n* (%)*	Male, *n* (%)*	*P*-value^#^
DM can lead to stroke.	188 (51.1%)	131 (69.6%)	57 (30.4%)	0.254
DM can lead to heart disease.	261 (70.9%)	184 (70.5%)	77 (29.5%)	0.232
DM can lead to loss of vision.	309 (84%)	230 (74.4%)	79 (25.6%)	0.035
DM can lead to renal disease.	295 (80.2%)	213 (72.2%)	82 (27.8%)	0.946
DM can lead to sexual impairment.	254 (69.0%)	176 (69.3%)	78 (30.7%)	0.056
DM can lead to peripheral neuropathy.	277 (75.3%)	199 (71.8%)	78 (28.2%)	0.741
DM can lead to amputation.	314 (85.3%)	252 (80.3%)	62 (19.7%)	0.114
DM can lead to poor wound healing.	346 (94%)	251 (72.5%)	95 (27.5%)	0.658
DM can lead to dental problems.	277 (75.0%)	210 (75.8%)	67 (24.2%)	0.008

Based on the statistical analysis results in Table 4, there were no significant differences between male and female patients in their level of knowledge regarding complications of DM, including hypo- and hyperglycemia. A total of 277 (75.3%) respondents knew common complications, exactly 326 (88.59%) knew about hypoglycemia as a DM complication, and 310 (84.2%) knew about hyperglycemia.

**Table 4:** Level of general knowledge of DM complications among the participants, categorized by gender. ^*^Data have been presented as *n* (%). The chi-square test *P*-value is considered significant if *P *<  0.05 DM, diabetes mellitus

DM complication awareness	Total, *n* (%)	Female, *n* (%)	Male, *n* (%)	*P*-value*
Knowledge of common complications of diabetes mellitus				0.752
I know	277 (75.3%)	199 (71.8%)	78 (28.2%)	
I don’t know	91 (24.7%)	67 (73.6%)	24 (26.4%)	
Knowledge of hypoglycemia Diabetes Mellitus Complications				0.964
I know	326 (88.59%)	236 (72.4%)	90 (27.6%)	
I don’t know	42 (11.41%)	30 (71.4%)	12 (28.6%)	
Knowledge of hyperglycemia diabetes mellitus complications				0.275
I know	310 (84.2%)	220 (71.0%)	90 (29.0%)	
I don’t know	66 (15.8%)	46 (69.7%)	12 (30.3%)	

## Discussion

The key findings from this study are that the average age of participants was 39.17 years, with females being slightly older (39.74 years) compared to males (37.75 years). This indicates that the study had a good representation in terms of age distribution. A total of 221 (60.1%) participants had a university degree or higher. On the other hand, the participants had a diverse range of occupations; exactly 122 (33.2%) respondents were employed. Other occupation categories included a total of 18 (4.9%) freelancers, 58 (15.8%) retirees, 79 (21.5%) students, and 91 (24.7%) unemployed individuals. These findings suggest diverse occupational backgrounds among the participants. According to the association between gender and the demographic information of the participants, there were significant differences in age, marital status, and occupation (*P* < 0.05). These findings were consistent with Gautam et al.'s study results, which found that the majority of patients with diabetes in Nepal had good knowledge about diabetes. They were aware of the risk factors, complications, and self-management practices [12].

In addition, the majority of the participants (144, 39.1%) had been living with diabetes for more than 10 years, followed by 23.1% (85) who had been diagnosed within the last year. This suggests that the study population consisted of individuals with varying levels of experience in terms of managing their diabetes. A significant number of participants (239, 64.9%) reported having a family history of diabetes. This indicates that genetics may play an essential role in the development of diabetes within this population. On the other hand, the majority of participants (247, 67.1%) reported that their doctor had discussed diabetes complications with them. This suggests that healthcare providers actively discuss potential complications with their patients, which is crucial in terms of diabetes management and complications prevention. The most reported sources of diabetes complication information were from diabetologists (131, 35.6%), followed by social media (81, 22%), relatives (65, 17.7%), and family medicine doctors (39, 10.6%). This indicates that individuals were getting information from various sources. These findings align with the study by Ghannadi et al., which demonstrated that knowledge, attitude, and practice all had a significant positive effect on self-management in type 2 diabetic patients on dialysis [13].

The findings of the study show that a majority of the participants have a good understanding of the various complications of DM. Specifically, 309 (84%) participants comprehend that DM can lead to the loss of vision, 295 (80.2%) are aware of the risk of renal disease, and 261 (70.9%) know about the association with heart disease. Additionally, 188 (51.1%) participants understand the association with stroke, 254 (69%) are aware of the potential for sexual impairment, and exactly 277 (75.3%) understand the risk of peripheral neuropathy. In terms of gender differences, a higher percentage of female participants have a good understanding of most complications compared to male participants. These gender differences were statistically significant for loss of vision (*P* = 0.035) and dental problems (*P* = 0.008). Overall, the findings suggest that the participants generally had good knowledge of the complications associated with DM, with female participants having a better understanding of some areas. These findings were consistent with Ng et al.’s study results, which found that patients with diabetes had a significant level of knowledge about their condition. The participants had basic knowledge about diabetes and its management [14].

Finally, regarding the knowledge of common complications of DM, the results indicate that a total of 277 (75.3%) participants were aware of them. Among females, 71.8% (199) knew about these complications, while 28.2% (78) of the males were aware of them. However, the difference in knowledge between males and females was not statistically significant (*P* = 0.752). Regarding knowledge of hypoglycemia as a complication of DM, 326 (88.59%) participants were aware of it. Among females, 236 (72.4%) were aware of hypoglycemia, while 27.6% (90) of the males were aware of it. Similarly, the difference in knowledge between males and females was not statistically significant (*P* = 0.964). Finally, in terms of knowledge of hyperglycemia as a complication of DM, 310 (84.2%) patients were aware of it. Among females, 220 (71.0%) were aware of hyperglycemia, while 90 (29.0%) males were aware of it. The difference in knowledge between males and females was also not statistically significant (*P* > 0.05). These findings aligned with the results of the study by Feleke et al., which suggested that tailored interventions should be developed to address the specific needs of different patient groups [15].

In general, this study shows that the studied population has a relatively good knowledge of diabetic complications, but some still lack knowledge. The results also indicate that individuals with diabetes receive information about diabetes complications from different sources, with healthcare providers and dialectologists playing a crucial role. However, some individuals turn to social media for information. Therefore, the study emphasizes the need for targeted education and awareness campaigns to ensure that people with diabetes can obtain accurate and reliable information from diverse sources.

This study had some limitations. The study primarily focused on Saudi individuals, so the findings may not apply to other populations with different cultural, social, and healthcare contexts. This limits the generalizability of the results. Another significant limitation in the current research is the cross-sectional design that gives only a snapshot of the levels of knowledge about diabetes complications among the surveyed individuals. Therefore, future research should be longitudinal and attempt to assess the link between better knowledge of diabetes complications and their management.

## Conclusions

The study concludes that while the studied population had a relatively good knowledge of diabetic complications, some still lacked knowledge. The study also supports the need for individuals with a family history of diabetes to be aware of their risk and take steps for prevention, as a large proportion of participants reported having a family history of diabetes. Furthermore, the study indicated active communication by the doctor about potential complications of diabetes, diabetes management, and prevention. Notably, 22% of information on diabetes complications comes from social media. Therefore, it is crucial to emphasize the importance of focused education and awareness actions to ensure that people with diabetes have access to reliable information from various sources. Future research should aim to assess the preventability of diabetes complications by improving knowledge through longitudinal studies.
